# Impact of Mask Type as Training Target for Speech Intelligibility and Quality in Cochlear-Implant Noise Reduction

**DOI:** 10.3390/s24206614

**Published:** 2024-10-14

**Authors:** Fergal Henry, Martin Glavin, Edward Jones, Ashkan Parsi

**Affiliations:** 1 Department of Computing and Electronic Engineering, Atlantic Technological University, Ash Lane, F91YW50 Sligo, Ireland; 2 Electrical and Electronic Engineering, University of Galway, University Road, H91TK33 Galway, Ireland; martin.glavin@universityofgalway.ie (M.G.); edward.jones@universityofgalway.ie (E.J.); ashk.parsi@gmail.com (A.P.)

**Keywords:** cochlear implant (CI), speech enhancement (SE), noise reduction (NR), speech intelligibility (SI), speech quality (SQ), machine learning (ML), neural network (NN), acoustic features, time–frequency (T-F), mask

## Abstract

The selection of a target when training deep neural networks for speech enhancement is an important consideration. Different masks have been shown to exhibit different performance characteristics depending on the application and the conditions. This paper presents a comprehensive comparison of several different masks for noise reduction in cochlear implants. The study incorporated three well-known masks, namely the Ideal Binary Mask (IBM), Ideal Ratio Mask (IRM) and the Fast Fourier Transform Mask (FFTM), as well as two newly proposed masks, based on existing masks, called the Quantized Mask (QM) and the Phase-Sensitive plus Ideal Ratio Mask (PSM+). These five masks are used to train networks to estimate masks for the purpose of separating speech from noisy mixtures. A vocoder was used to simulate the behavior of a cochlear implant. Short-time Objective Intelligibility (STOI) and Perceptual Evaluation of Speech Quality (PESQ) scores indicate that the two new masks proposed in this study (QM and PSM+) perform best for normal speech intelligibility and quality in the presence of stationary and non-stationary noise over a range of signal-to-noise ratios (SNRs). The Normalized Covariance Measure (NCM) and similarity scores indicate that they also perform best for speech intelligibility/gauging the similarity of vocoded speech. The Quantized Mask performs better than the Ideal Binary Mask due to its better resolution as it approximates the Wiener Gain Function. The PSM+ performs better than the three existing benchmark masks (IBM, IRM, and FFTM) as it incorporates both magnitude and phase information.

## 1. Introduction

Typically, in a mask-based supervised speech separation system, acoustic features provide the input to a learning machine while a suitable mask serves as the output, with a suitable target mask being used during training. Such a system can be used to perform speech enhancement (otherwise known as noise reduction) in a range of applications. The particular application of interest in this paper is a cochlear implant, which is a prosthesis used to restore hearing to people with hearing loss due to damage incurred by hair cells in the cochlea. Users of cochlear implants can understand the majority of what a speaker has to say in quiet environments. This is essentially termed speech intelligibility. However, they struggle to understand what is being said when there is interfering background noise. Previous studies have applied neural networks [1,2] and recurrent neural networks [3] to improve speech intelligibility in noise for users of cochlear implants.

The works of Chen and Wang [4,5] provide a good overview of DNN-based mask estimation for supervised speech separation. They introduce masks such as the Ideal Binary Mask (IBM), Target Binary Mask (TBM), Ideal Ratio Mask (IRM), Fast Fourier Transform Magnitude (FFT-MAG), Gammatone Frequency Power Spectrum (GF-POW), Phase-Sensitive Ideal Ratio Mask (PSIRM), and Complex Ideal Ratio Mask (cIRM). Michelsanti et al. [6] also provide an overview of deep-learning-based audiovisual speech enhancement and separation. When dealing with training targets, they discuss direct mapping, mask approximation, and indirect mapping. Wang [7] proposed a deep neural network for time-domain signal reconstruction, and in that study, both the IBM and the IRM were utilized. Nossier et al. [8] performed a comparison of mapping and masking targets (including some of the aforementioned masks) for various deep-learning-based speech enhancement architectures. Samui et al. [9] employed the IRM, PSM (Phase-Sensitive Mask), and cIRM when performing speech enhancement using a fuzzy deep belief network. 

While previous work has evaluated a wide variety of masks, based on both magnitude and phase information, there is scope for further improvement. The motivation for this paper is to extend previous work by proposing two new masks, one an adaptation of the IBM/IRM, and therefore using primarily magnitude information, with the second an adaptation of the PSM, also making use of phase information. The paper also reports an experimental study on these new masks, comparing them to some benchmark masks from the literature.

This paper is organized as follows. The literature pertaining to previous work in the field of binary, ratio, and phase mask estimation and speech enhancement is summarized in Section 2. Section 3 outlines the mathematical definitions of existing masks and new masks proposed in this study. Section 4 covers the methodology for this study, including the dataset used; the masks under evaluation, including the newly proposed masks; the machine learning architecture used; the cochlear implant simulation; and the metrics used for predicting intelligibility, quality, and similarity. The experimental results are contained in Section 5. Section 6 discusses the ensuing results and Section 7 makes some concluding remarks.

The key contributions of this paper are as follows:

- It proposes the use of a new Quantized Mask (QM), which is an approximation of the Wiener Gain Function (WGF), for the application of speech enhancement in cochlear implants;
- It also proposes another new mask called the PSM+, which is a hybrid of the PSM and the IRM for the same purpose;
- It compares the performance of these masks to other well-established masks (e.g., IBM, IRM, and FFTM) in terms of speech intelligibility and quality for normal hearing as well as speech intelligibility and similarity for hearing impairment.

## 2. Background

A mask is essentially a two-dimensional array of values where each horizontal row is composed of overlapping time frames of a signal and each vertical column is composed of its corresponding frequency components. This is referred to as the time–frequency representation of a signal and the entries in the array are referred to as time–frequency units. According to Lee et al. [10], there are two main learning schemes for time–frequency masks, namely mask approximation (MA) and spectra approximation (SA). The former approach minimizes the MA objective function while the latter approach minimizes spectral distortion. 

A widely used mask in speech enhancement is the Ideal Binary Mask (IBM). Heymann et al. [11] performed neural-network-based spectral mask estimation for beamforming using this mask. In their work, they defined both an ideal binary mask for noise (IBM_N_) as well as an ideal binary mask for the target signal (IBM_X_). Kjems et al. [12] investigated the role of the mask pattern in the intelligibility of ideal binary-masked speech. They also defined the target binary mask (TBM) as that obtained by comparing the target energy to that of a speech-shaped noise (SSN) reference signal matching the long-term spectrum of the target speaker in each time–frequency (T-F) unit.

Others have focused on ratio masks and adaptations thereof. Abdullah et al. [13] employed a Quantized Correlation Mask to improve the efficiency of a DNN-based speech enhancement system. This is a correlation-based learning target where the IRM is optimized by adjusting inter-channel correlation factors. Bao and Abulla [14] also exploited normalized cross correlation (NCCC) when developing a noise masking method based on estimating a ratio mask using channels based on the Gammatone frequency scale. Previous work in the area of masking and correlation can be found in the work of Bao et al. [15,16]. More recently, Lang and Yang [17] trained a Corrected Ratio Mask (CRM) with deep neural networks for echo cancellation in laser monitoring signals.

Much of the work using deep learning networks for speech enhancement has primarily focused on estimating the magnitude spectrum while reusing the phase from the noisy speech for resynthesis. However, some studies have also endeavored to include phase-related information when generating masks. Choi et al. [18] used phase information and proposed a polar-coordinate-wise complex-valued masking method to implement the Complex Ideal Ratio Mask. The phase-sensitive filter (mask) was employed by Erdogan et al. [19] to perform speech separation using deep recurrent neural networks. Hasannezhad et al. [20] performed speech enhancement by estimating a phase-sensitive mask using a novel hybrid neural network. A bounded approximation of the Phase-Sensitive Mask (PSM) was approximated by Lee et al. [10]. They then used a joint learning algorithm that trains the approximated value through its parameterized variables of speech magnitude spectra, noise magnitude spectra, and the phase difference between clean and noisy spectra. Finally, they used a warping function to control the dynamic range of the magnitude spectra. Li et al. [21] compared the PSM to the IRM and obtained improved performance for speech enhancement by focusing on smaller values using relative loss. 

Mayer et al. [22] also considered the impact of phase estimation when using time–frequency masking to perform single-channel speech separation. Indeed, a fully convolutional neural network was used by Ouyang et al. [23] to demonstrate that complex spectrogram processing is effective at phase estimation, resulting in the improved reconstruction of clean female speech. Tan and Wang [24] proposed a convolutional recurrent network (CRN) for complex spectral mapping in speech enhancement. Complex spectral mapping estimates the real and imaginary spectrograms of clean from noisy speech. It aims to enhance both magnitude and phase responses of noisy speech. A PSM and conditional generative adversarial network (cGAN) architecture was proposed by Routray and Mao [25] and showed that it performed better than other baseline networks in terms of speech quality and intelligibility. Wang and Bao [26] also incorporated phase information when estimating masks for speech enhancement. 

Zhang et al. [27] proposed a time–frequency attention (TTF) module and used a residual temporal convolutional network (resTCN) to perform monoaural speech enhancement with IRM and PSM training targets. A phase-aware speech enhancement using a deep neural network was also proposed by Zheng et al. [28]. During training, they transformed the unstructured phase spectrogram to its derivative with respect to time (known as instantaneous frequency deviation (IFD)). In testing, a post-processing method was proposed to recover the phase spectrogram from the estimated IFD. Sivapatham et al. [29,30] combined the phase and correlation in a mask to improve speech intelligibility in a deep neural network. 

In this paper, perfect masks that are generated to investigate their oracle performance will be referred to as ideal masks. These masks are not estimated and are generated with perfect knowledge of the clean speech signal and noise. Masks that are generated using a neural network will be referred to as estimated masks. 

## 3. Masks

A number of masks feature prominently in the existing literature. Five such masks are defined in Section 3.1, namely the IBM, IRM, FFTM, PSM (ORM), and cIRM. Two new masks are newly proposed in this study, namely the QM and PSM+, and are defined in Section 3.2 accordingly. Some visual examples of the masks are provided in Section 3.3. 

### 3.1. Existing Masks

The first mask considered is the well-established Ideal Binary Mask (IBM) [31], which is defined in Equation (1) as follows:

(1) $$IBM=\left\{\begin{array}{c} 1,\;if\;SNR\left(t,f\right)>LC\\ 0,\;otherwise \end{array}\right.$$

Here, *SNR*(*t*,*f*) is the local signal-to-noise ratio within a time–frequency unit. To preserve sufficient intelligibility, the local criterion (*LC*) is usually chosen to be 5 dB lower than the SNR of the mixture [32]. It is referred to as a hard mask as it has hard values of 0 and 1.

Another mask widely used in speech separation is the Ideal Ratio Mask (IRM) [33]. It is defined in Equation (2) as follows:

(2) $$IRM=\sqrt{\frac{{S(t,f)}^{2}}{{S(t,f)}^{2}+{N(t,f)}^{2}}}$$

Here, *S*(*t*,*f*)^2^ and *N*(*t*,*f*)^2^ are the speech energy and noise energy within a time–frequency unit. This mask is referred to as a soft mask and can have any value in the range of [0, 1].

The Spectral Magnitude Mask [32], referred to here as the FFT-MASK (FFTM), is defined in Equation (3) as follows:

(3) $$FFTM=\frac{\left|S(t,f)\right|}{\left|Y(t,f)\right|}$$

This is the ratio of the spectral magnitudes of the clean and noisy speech. Unlike the previous two masks, it is unbounded in the positive direction, i.e., its values are in the range of [0, *∞*].

While the majority of masks in the literature are based solely on magnitude information, several researchers have also proposed masks that incorporate phase information. For example, the Phase-Sensitive Mask (PSM) [19] is defined in Equation (4) as follows:

(4) $$PSM=\frac{\left|S(t,f)\right|}{\left|Y(t,f)\right|}\cos\theta$$

Here, *S*(*t*,*f*) and *Y*(*t*,*f*) are the clean and noisy spectra, respectively, while *θ* represents the phase difference between the clean and the noisy speech within a time–frequency unit. The PSM is unbounded in both negative and positive directions, i.e., its values are in the range of [−*∞*, *∞*]. It was also defined by Wang et al. [34] using Equation (5), where, again, *S* denotes the clean speech signal, *Y* is the noisy speech signal, *Re* denotes the real part, *θ_S_* is the phase of the clean speech, *θ_Y_* is the phase of the noisy speech, and the (*t*,*f*) notation has been omitted for simplicity.

(5) $$PSM=\frac{\left|S\right|}{\left|Y\right|}cos\left(\theta_{S}-\theta_{Y}\right)=Re\left(\frac{S}{Y}\right)$$

The spectrum of the clean speech can be represented in complex rectangular form in Equation (6) where subscript *r* is the real part, subscript *i* is the imaginary part, and $j=\sqrt{-1}$.

(6) $$S=S_{r}+jS_{i}$$

Similarly, the spectrum of the noise *N* can be represented using Equation (7).

(7) $$N=N_{r}+jN_{i}$$

Assuming that the noise is additive, the spectrum of the noisy speech can be written in complex rectangular form as in Equation (8).

(8) $$Y=S+N=\left(S_{r}+N_{r}\right)+j\left(S_{i}+N_{i}\right)$$

Combining Equations (5) and (8) results in Equation (9).

(9) $$PSM=Re\left(\frac{S_{r}+jS_{i}}{\left(S_{r}+N_{r}\right)+j\left(S_{i}+N_{i}\right)}\right)$$

This can be expanded to produce Equation (10).

(10) $$PSM=\frac{\left(S_{r}^{2}+S_{i}^{2}\right)+\left(S_{r}N_{r}+S_{i}N_{i}\right)}{\left(S_{r}^{2}+S_{i}^{2}\right)+\left(N_{r}^{2}+N_{i}^{2}\right)+2\left(S_{r}N_{r}+S_{i}N_{i}\right)}$$

Finally, using * to represent the complex conjugate, this equation can be further simplified to Equation (11), which demonstrates, analytically, that the PSM is equivalent to the Optimal Ratio Mask (ORM). The ORM was derived by Liang et al. [35] to maximize the signal-to-noise ratio and was further used in speech separation in [36,37].

(11) $$PSM=\frac{{\left|S\right|}^{2}+Re\left(SN^{*}\right)}{{\left|S\right|}^{2}+{\left|N\right|}^{2}+2Re\left(SN^{*}\right)}=ORM$$

The Complex Ideal Ratio Mask (cIRM) is well described in [38,39] and is defined mathematically in Equation (12) as follows:

(12) $$cIRM=M_{r}+iM_{i}$$

Here, *M_r_* and *M_i_* are the real and imaginary components of the mask and are defined in Equations (13) and (14):

(13) $$M_{r}=\frac{Y_{r}S_{r}+Y_{i}S_{i}}{Y_{r}^{2}+Y_{i}^{2}}$$

(14) $$M_{i}=\frac{Y_{r}S_{i}-Y_{i}S_{r}}{Y_{r}^{2}+Y_{i}^{2}}$$

Here, *Y_r_* and *Y_i_* are the real and imaginary components of the spectrum of the noisy speech while *S_r_* and *S_i_* are the real and imaginary components of the spectrum of the clean speech. As stated in [38], *M_r_* is theoretically identical to the PSM. *M_i_* is unbounded in both negative and positive directions, i.e., its values are in the range of [−*∞*, *∞*].

### 3.2. Proposed Masks

Two new masks are proposed in this paper. The first mask is inspired by the IBM but has multiple local SNR thresholds rather than just one. In essence, it is a quantized approximation to the Wiener Gain Function (WGF) [40,41] and hence is referred to here as the Quantized Mask (QM). The number of local criteria and their respective values have been derived empirically to optimize the STOI score, and the mask is defined in Equation (15) as follows:

(15) $$QM=\left\{\begin{array}{c} 0,\;if\;SNR\left(t,f\right)<LC1\\ 0.25,\;if\;LC1>SNR\left(t,f\right)\leq LC2\\ 0.5,\;if\;LC2>SNR\left(t,f\right)\leq LC3\\ 0.75,\;if\;LC3>SNR\left(t,f\right)\leq LC4\;\\ 1,\;if\;SNR\left(t,f\right)>LC4 \end{array}\right.$$

**Figure 1:**
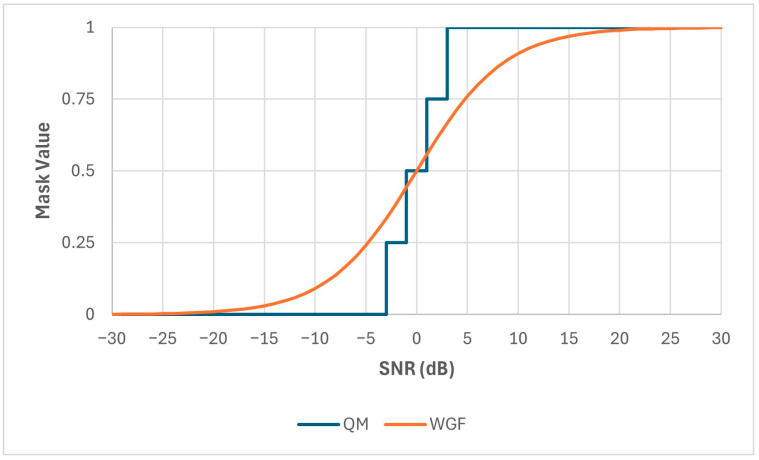
Quantized Mask (QM) and Wiener Gain Function (WGF) at mixture SNR of 5 dB.

If *SNR* is the signal-to-noise ratio of the mixture, then *LC*1 = *SNR*-8, *LC*2 = *SNR*-6, *LC*3 = *SNR*-4, and *LC*4 = *SNR*-2. For example, the *QM* for a mixture SNR of 5 dB is illustrated in Figure 1. The motivation for using this mask is to provide more granular magnitude information than the IBM and hence maximize speech intelligibility.

The second new mask proposed in this paper is a hybrid mask as defined in Equation (16) and is referred to as the *PSM+*. It uses Equation (4) to calculate modified PSM values:

(16) $$PSM+=\left\{\begin{array}{c} IRM\left(t,f\right),\;if\;PSM\left(t,f\right)<0\\ PSM\left(t,f\right),if\;0\geq PSM(t,f)\leq 2\;\\ 2,\;if\;PSM(t,f)>2 \end{array}\right.$$

As can be seen in Equation (16), if the PSM values are negative, they are replaced by their corresponding IRM values in Equation (2). Based on the results of the empirical results in this study, if they exceed a value of 2, they are clipped at 2. This approach is motivated by the work of Zhou et al. [42], who employed mask fusion with multi-target learning for speech enhancement. Because the end goal of this research is to optimize speech intelligibility, the retention of as much magnitude and phase information as possible is beneficial. When the PSM+ is used, approximately half of the mask values (i.e., those PSM values that are positive) exploit both magnitude and phase. Rather than discarding the remaining negative PSM values and treating them as zero, it was decided to use their corresponding IRM values, thus retaining additional magnitude information.

### 3.3. Examples

**Figure 2:**
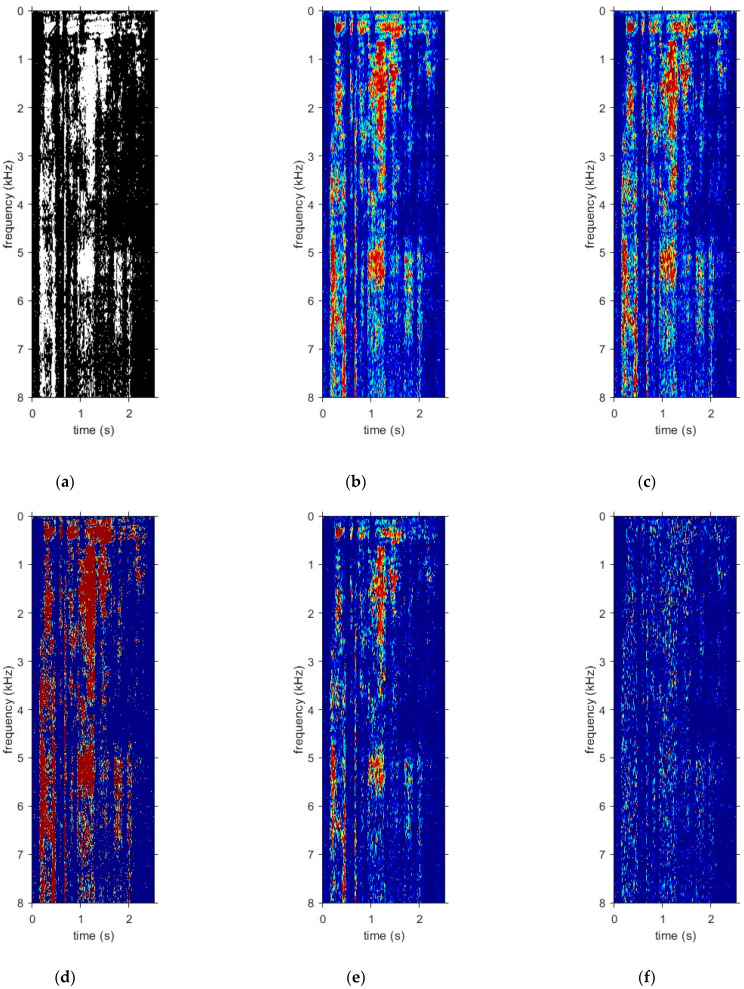
Ideal masks for the utterance “choose between the high road and the low” mixed with babble noise at −5 dB SNR: (**a**) IBM; (**b**) IRM; (**c**) FFTM; (**d**) QM, (**e**) cIRMr; (**f**) cIRMi.

By way of example, Figure 2 shows color images of the ideal masks for a particular utterance mixed with babble noise at −5 dB SNR. The utterance was taken from the IEEE-Harvard corpus [43] and sampled at 16 kHz. A grayscale is used in the figure, with white pixels denoting a value of 1 and black pixels denoting a value of 0. Although they are not identical, broadly speaking, the same pattern is more or less evident in the masks labelled (a) to (e). However, for the mask labelled (f), which is the imaginary part of the cIRM, the pixels are predominantly black, suggesting that most mask values are closer to 0 (noise-dominated). 

## 4. Materials and Methods

This section initially outlines the dataset used for the experimental work in this study. It then proceeds to discuss how the masks presented in Section 3 are used as training targets, including both well-established masks as well as two newly proposed masks. Following this, it provides an overview of the machine learning network used to estimate the masks. It then discusses how a vocoder is used to simulate the operation of a cochlear implant. Then, the objective metrics are explained, and these are used to determine the performance of the estimated masks in terms of speech intelligibility, quality, and similarity. Finally, all software sources are documented.

### 4.1. The Dataset

The IEEE-Harvard corpus [43] was selected as the dataset for the clean speech utterances for this study. All 720 sentences were produced by a male speaker, with 600 of these used for training purposes (including generating ideal masks) and 120 used for testing purposes. This dataset was chosen as it has been widely used in other related studies such as in [1,44], which makes it is useful for comparison. The sentences themselves are phonetically balanced and the phonemes have been specifically selected for their frequency content. Three noise sources were used in the initial part of this study, i.e., to evaluate the oracle performance. These noises were babble, factory, and speech-shaped noise (SSN). The babble was produced by recording 100 people speaking in a canteen while the factory noise was from a car production hall. These first two noise sources were taken from the Signal Processing Information Base (SPIB) [43] while the third was generated by mixing white noise from SPIB with the long-term speech envelope of the IEEE-Harvard speech corpus. The factory noise was useful when comparing initial results to those in previous work such as [5,32]. Only babble and SSN were used in association with the neural networks to generate estimated masks as these were representative of non-stationary and stationary noise, respectively. The training and test mixtures were generated in accordance with the work described in [32]. Random cuts from the first 2 min of the babble and SSN were mixed with the training utterances at −5 dB and 0 dB SNR. Similarly, random cuts from the last 2 min of both noises were mixed with the test utterances at −5 dB, 0 dB, and 5 dB SNR.

### 4.2. Mask Generation

An FFT of length 1024 was used to estimate the ideal masks for testing the oracle performance while an FFT of length 320 was used for the estimated masks. Five different estimated masks were evaluated for this study. Three of these were from the existing literature, namely IBM, IRM, and FFTM. The other two were the masks proposed in Section 3.2, namely QM and PSM+.

As previously stated, the estimated masks were generated by the neural network. Although the ideal IBM in Equation (1) is a hard mask, its estimated version is a soft mask. It is straightforward to calculate the IRM using Equation (2) while the FFTM is calculated using Equation (3). However, based on empirical testing, all values above 1.5 were clipped at 1.5 in this study. This was used rather than using log compression suggested in [32] or hyperbolic tangent compression suggested in [38,45]. The proposed QM was calculated using Equation (9). Finally, the second newly proposed mask in this study (PSM+) was calculated using Equation (10). Due to its unbounded nature, the cIRM was not estimated in this study. However, as previously stated, the real part of the cIRM is indeed the PSM.

In each case, the estimated speech spectrum $\hat{S}$ is calculated using Equation (17) as follows, where *M_est_* is the estimated mask, *Y* is the noisy spectrum, and *θ_Y_* is the phase of the noisy speech.

(17) $$\hat{S}=M_{est}.\left|Y\right|e^{j\theta_{Y}}$$

The one exception to this is the complex ratio mask where *, in this case, represents complex multiplication in Equation (18). However, this is only used during oracle performance experiments.

(18) $$\hat{S}=M_{est}*Y$$

### 4.3. The Neural Network

**Figure 3:**
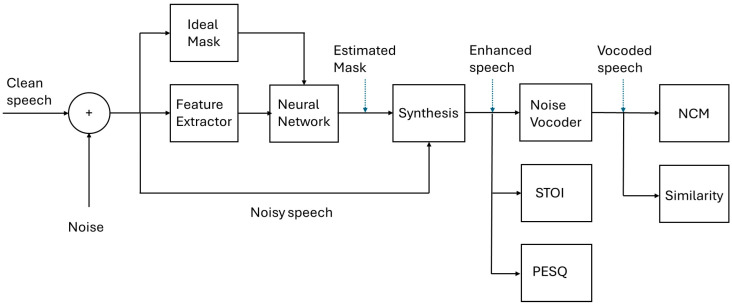
Block diagram of the test system.

The neural network is an integral part of the test system in Figure 3 and is based on the multilayer perceptron (MLP) architecture. Although MLP is a basic architecture, it is considered to be a well-known and acceptable benchmark based on previous research in the field. Following on from the work of [46], 24 Mel Spectrogram acoustic features were extracted at the frame level and used as the inputs. The sampling frequency was 16 kHz and the analysis window was 20 ms with 50% overlap. The features were concatenated with delta features and then smoothed using an auto-regressive moving average filter. Three hidden layers were used, with each layer containing 1024 nodes. The sigmoid activation function was used for hidden layers. It was also used for the output layer when the output was in the range of [0, 1], with the linear activation function being used otherwise. The standard backpropagation algorithm was used without dropout. Adaptive gradient descent was used as the optimization algorithm with a scaling factor of 0.0015. The number of training epochs used was 20. Momentum was initially set to 0.5 and changed to 0.9 after epoch 5. The mean squared error (MSE) was used as the cost function to predict the error between the predicted and expected output values. The outputs were the mask values, which had dimensions of 320, corresponding to the number of frequency bins. The outputs were spliced into a 5-frame window according to [32]. Ideal masks were used as the training targets and the estimated masks were used during testing. No pretraining was used during these experiments. This neural network can be used to estimate masks as per the study of Healy et al. [47] and these masks can be used in a speech segregation system.

### 4.4. Vocoder Simulation

**Figure 4:**
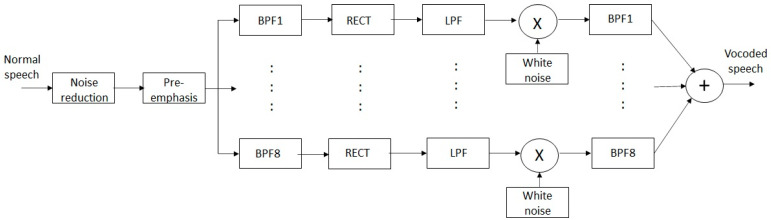
Block diagram of 8-channel noise vocoder.

Channel vocoders have been widely used to simulate speech processing in cochlear implants, as described by Cychosz et al. [48]. For the purposes of this study, an eight-channel noise vocoder (see Figure 4) was used for CI simulation. The pre-emphasis filter had a cutoff frequency of 2 kHz and a roll-off of 3 dB/octave. The band pass filters (BPFs) in the eight channels had center frequencies of 366, 526, 757, 1089, 1566, 2252, 3241 and 4662 Hz. The low pass filters (LPFs), which follow the rectification stage (RECT), had a cutoff frequency of 120 Hz. The noise vocoder was used to vocode the normal speech so that it mimicked the sound heard by the wearer of a cochlear implant.

### 4.5. Metrics

Objective speech quality and intelligibility metrics can be divided into two classes, namely intrusive metrics, which use a reference signal, and nonintrusive metrics, which do not use a reference signal. Falk et al. [49] investigated twelve such metrics for predicting objective quality and intelligibility for users of hearing aids and cochlear implants. This followed on from the work of Cosentino et al. [50], Santos et al. [51], Kokkinakis and Loizou [52], and Hu and Loizou [53]. 

Short-Time Objective Intelligibility (STOI) [54] is used here as a speech intelligibility metric for normal hearing, i.e., speech signals that have not been vocoded. It measures the correlation between the short-time temporal envelopes of a clean utterance and an estimated utterance: in this study, one that was generated via noise reduction conducted on a noisy signal. The STOI varies from 0 to 1 and can be interpreted as 0% to 100% intelligibility. It is defined in Equation (19), where *d_j_*(*m*) is an intermediate intelligibility measure defined in [55], *M* is the total number of frames, and *J* is the number of one-third octave bands used for analysis.

(19) $$STOI=\frac{1}{JM}\sum_{j,m}d_{j}(m)$$

Perceptual Evaluation of Speech Quality (PESQ) [56] is used here as a speech quality metric for normal hearing. It utilizes an auditory transform to generate a loudness spectrum. The loudness spectra of clean utterances are compared to those of estimated utterances, with the score ranging from −0.5 to 4.5, to predict mean opinion score (MOS). In this paper, the MOS-mapped score according to ITU P.862.2 is used [57]. PESQ can be calculated using Equation (20), where *D_ind_* is the disturbance value, *A_ind_* is the asymmetrical disturbance value, *a*_0_ = 4.5, *a*_1_ = −0.1, and *a*_2_ = −0.039.

(20) $$PESQ=a_{0}+a_{1}D_{ind}+a_{2}A_{ind}$$

If the raw model PESQ output is *PESQ_r_*, the output mapping function used in the wideband extension is defined in Equation (21).

(21) $${PESQ}_{MOS}=0.999+\frac{4.999-0.999}{1+e^{-1.3669{PESQ}_{r}+3.8224}}$$

The Normalized Covariance Measure (NCM) [58] is used to predict speech intelligibility for hearing-impaired listeners such as those wearing cochlear implants. Based on the work of Lai et al. [59], it is used to predict the intelligibility of vocoded and wideband speech. It is a Speech Transmission Index (STI) measure and is based on the covariance of the envelopes between clean vocoded speech and estimated vocoded speech. NCM scores range from 0 (0%) to 1 (100%) intelligibility. It is defined in Equation (22), where *W_i_* is the band importance weight applied to each of the *k* bands and *TI_i_* is the transmission index in each band.

(22) $$NCM=\frac{\sum_{i=1}^{k}W_{i}{TI}_{i}}{\sum_{i=1}^{k}W_{i}}$$

Euclidean distance has been used in various audio applications such as in measuring the similarity of a speaker’s voice characteristics [60], performing speech recognition [61], and measuring the similarity between two songs [62]. Therefore, it was decided to use a final group of measures to estimate the similarity between clean vocoded speech and estimated vocoded speech. Histograms were generated (using Scott’s Rule for the optimum number of bins) for clean, mixture (i.e., noisy), and estimated vocoded signals. These were normalized to represent probability distribution functions (pdfs) and the distance/similarity between various pdfs was measured. All metrics were defined and categorized by Cha in [63]. For this study, only distances were used, where similarity (*s*) was related to distance (*d*) in Equation (23) as follows:

(23) s=1−d

As outlined in [63], eight different families were used to measure similarity. Euclidean *L*_2_ and Chebyschev *L_∞_* similarities were calculated for the *L_p_* Minkowski family. Sorensen, Gower, and Soergel similarities were measured for the *L*_1_ family. Intersections, Wave Hedges, Czekanowsi, Motya, Ruzicka, and Tanimoto similarities were calculated for the Intersection family. Inner Product, Cosine, Kumar Hassebrook, and Dice similarities were calculated for the Inner Product family. Fidelity, Bhattacharya, Matusita, and Squared Chord similarities were calculated for the Fidelity family. Squared Euclidean similarity was calculated for the Squared *L*_2_ family. Taneja and Average similarities were calculated for the Combinations family. Kullback–Leibler Divergence was calculated for the Shannon’s Entropy family. The Kullback–Leibler Divergence was calculated using Equation (24) as follows:

(24) $$d_{KL}=\sum_{i=1}^{nbins}P_{i}ln\left(\frac{P_{i}}{Q_{i}}\right)$$

Here, *P_i_* and *Q_i_* are the pdf values in each histogram bin *i* and *nbins* is the number of bins. Care was taken to avoid the case of 0*ln*0.

Finally, a correlation coefficient was calculated to measure the correlation between pdfs. Its value could range from −1 (direct negative correlation) to 1 (direct positive correlation). If it was 0, there was no correlation.

### 4.6. Software

MATLAB R2023b was used for all experiments. Software for the neural network architecture and for calculating STOI scores was sourced at http://web.cse.ohio-state.edu/pnl/DNN_toolbox/ [32] (accessed on 5 April 2021) and adapted according to the needs of specific experiments. When conducting the oracle performance evaluations, software for computing the IBM was sourced at https://ch.mathworks.com/matlabcentral/fileexchange/33199-ideal-binary-mask (accessed on 3 March 2023). It was then modified to calculate the other masks used in this study. To simulate the behavior of a cochlear implant, the vocoder implementation at https://github.com/vmontazeri/cochlear-implant-simulation (accessed on 10 December 2020) was used. The software for calculating PESQ and NCM scores was sourced from the appendix of [44]. Software for calculating probability distribution functions and similarity was sourced at https://uk.mathworks.com/matlabcentral/fileexchange/97142-probability-density-functions-distance-and-similarity (accessed on 17 June 2024). 

## 5. Results

A feasibility study was conducted initially with ideal masks (i.e., “oracle” masks) in order to establish an upper limit on performance. Here, the 600 training utterances were used for measuring the oracle performance of the masks. Following on from this, the 120 test utterances were used for measuring how effective the neural networks were at estimating the masks and hence performing noise reduction on the mixtures. In all cases, the best (most effective) scores are shown here in bold text while the worst (least effective) scores are in italics. ‘Best’ means the highest mean/median and lowest standard deviation.

### 5.1. Oracle Performance

Oracle STOI scores for normal speech for each of the ideal masks evaluated are given in Table 1 in the form of mean, median, and standard deviation values. Three different noise types at −5 dB SNR have been used. The higher the mean/median is and the lower the standard deviation for STOI is, the better the intelligibility is. In all cases in Table 1, the median is slightly greater than the mean score. Regardless of the noise type, cIRM had a perfect mean score of 1 and a standard deviation of 0 and was able to perfectly recover the clean speech from the noisy mixture [34] and so has been excluded from Table 1. Of the other masks, the FFTM was the most effective mask with the highest mean and lowest standard deviation scores. The IBM was the least effective mask with the lowest mean and highest standard deviation scores.

**Figure 5:**
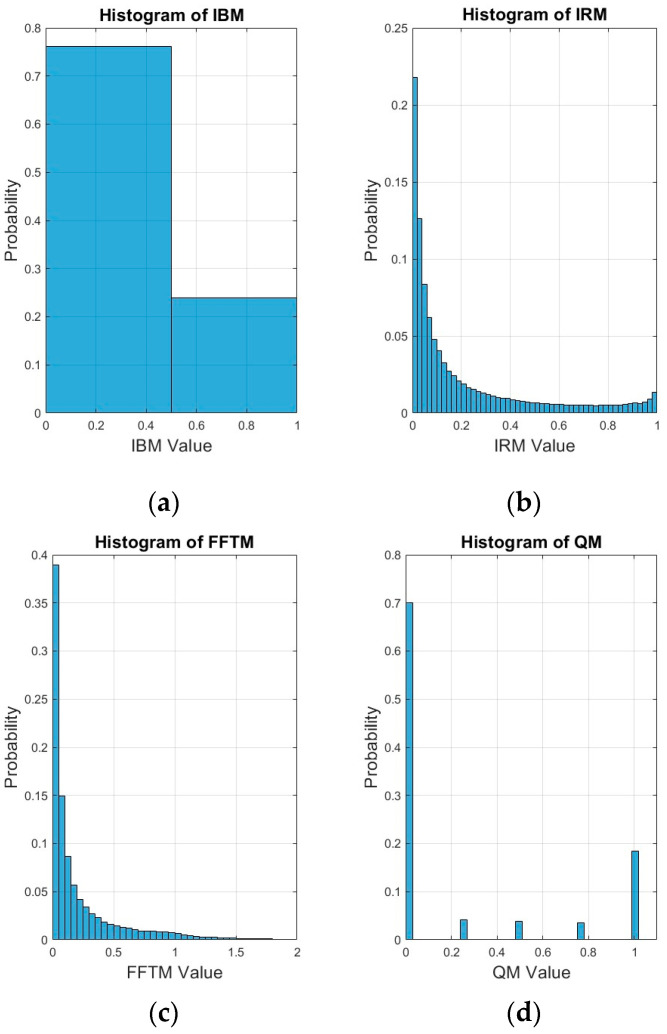

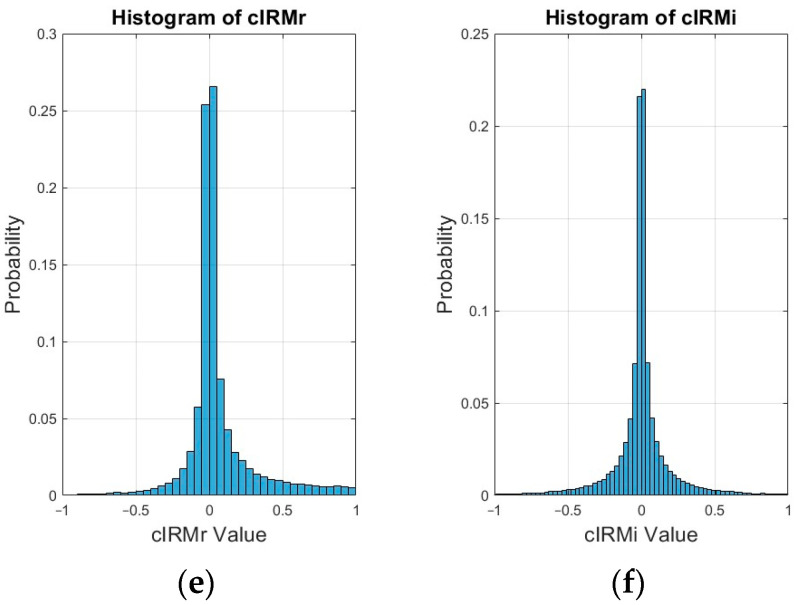
Histograms for the ideal masks for the utterance “choose between the high road and the low” mixed with babble noise at −5 dB SNR: (**a**) IBM; (**b**) IRM; (**c**) FFTM; (**d**) QM, (**e**) cIRMr; (**f**) cIRMi.

According to [38], targets/masks that take on values in the range of [0, 1] can be more conducive for supervised learning with deep neural networks. To better visualize the distribution of mask values, histograms were generated for the same sample utterance as in Figure 2 for each of the ideal masks. These histograms have been plotted in Figure 5 for babble noise at −5 dB SNR. This information is useful for deciding what values to clip for some of the unbounded mask values. It also provides insight on what percentage of negative mask values used the IRM and what percentage of positive mask values used the PSM when generating the PSM+. The experimental results in this study have demonstrated that clipping a small percentage of mask values is acceptable as speech intelligibility is still preserved. However, discarding a large percentage of mask values simply because they are negative has a detrimental effect on speech intelligibility as these mask values carry a significant amount of useful information. For the IBM, there are only two values, i.e., 0 and 1. A total of 24% of the values were equal to 1 (speech-dominant) while 76% of the values were 0 (noise-dominant) for babble noise. In the interests of brevity, histograms for factory noise and SSN have not been plotted in Figure 5. However, for comparison, for SSN, 15% of the values were equal to 1 while 85% of the values were equal to 0. For factory noise, 11% of the values were equal to 1 while 89% of the values were equal to 0. For the IRM, the values decayed approximately exponentially between 0 and 1. However, they rose slightly again as the IRM value reached 1. For the FFTM, the values also decayed exponentially. The maximum value was 74.6, with 94.5% of the values being in the range of [0, 1] (only values from 0 to 2 have been plotted in Figure 5c). A total of 97.9% of the values lay in the range of [0, 1.5]. For the QM, 70.05% of the values fell at quantization level 1, 4.13% fell into quantization level 2, 3.86% fell into quantization level 3, 3.55% fell into quantization level 4 and 18.41% fell into quantization level 5. For cIMRr, the minimum and maximum values were −72.7869 and 74.5591, respectively, and 56.1% of the values were in the range of [0, 1]. A total of 58.5% of the values were in the range of [0, 2]. A total of 41.1% of the values were negative. The mean and standard deviation were 0.0918 and 0.5871, respectively. For cIMRi, the minimum and maximum values were −45.8876 and 35.1535, respectively. A total of 49.3% of the values were in the range of [0, 1] while 50% of the values were in the range of [0, 2]. A total of 49.7% of the values were negative while the mean and standard deviation were −0.0005 and 0.4747, respectively. Both the cIMRr and the cIRMi distributions appeared to be reasonably symmetric around 0 but cIMRr was skewed slightly to the right.

### 5.2. Normal Hearing Speech Intelligibility

**Figure 6:**
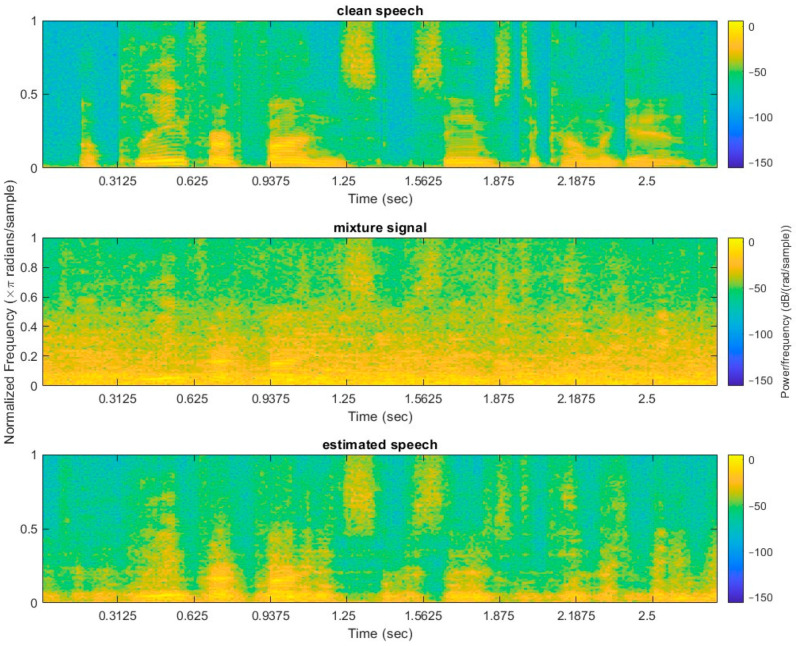
Spectrograms of normal clean speech, normal clean speech mixed with babble noise at −5 dB SNR, and IBM-estimated normal speech for the utterance “A plea for funds seems to come again”. Spectrograms use the Hanning Window with 20 ms frames and 50% overlap.

As an example of the impact of speech estimation, spectrograms from the experiments in this study have been illustrated in Figure 6 for a sample normal clean speech utterance, a mixture of clean speech and babble noise at −5 dB SNR, and the resulting speech signal synthesized using the estimated IBM. From Figure 6, it can be seen that the estimated speech (bottom of Figure 6) using the IBM bears a reasonable resemblance to the original clean speech (top of Figure 6); in particular, transient events are reasonably well preserved while harmonics are also reasonably well preserved.

Table 2 shows the STOI scores for the estimated masks for babble and SSN across a range of SNRs. It should be noted that all estimated masks performed well in terms of STOI improvement relative to the normal mixtures. The strongest-performing mask for normal speech intelligibility was the PSM+ while the least effective mask was the IRM. When averaging over the two noise types and the three SNRs, the PSM+ showed a 15.1% improvement while the IRM showed a 14.2% improvement. The FFTM performed best for babble noise while the PSM+ performed best for SSN.

### 5.3. Normal Hearing Speech Quality

Table 3 shows the PESQ scores for the estimated masks for babble and SSN across a range of SNRs. All estimated masks performed well in terms of PESQ improvement relative to the normal mixtures. The strongest performing mask for normal speech quality was the PSM+ while the least effective mask was the FFTM. When averaging over the two noise types and the three SNRs, the PSM+ showed a 27.7% improvement while the FFTM showed a 20.3% improvement. The PSM+ performed best for both babble noise and SSN and was closely followed by the QM.

### 5.4. Hearing-Impaired Speech Intelligibility

**Figure 7:**
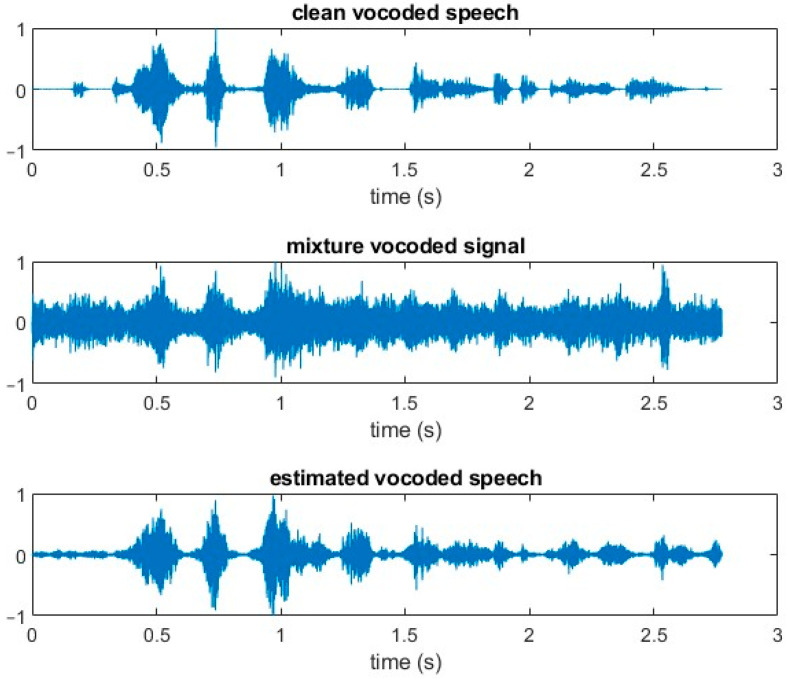
Time-domain representation of vocoded signals for clean speech, clean speech mixed with babble noise at −5 dB SNR, and IBM-estimated speech for the utterance “A plea for funds seems to come again”.

According to Newman and Chatterjee [64], noise-vocoded speech preserves the amplitude structure. However, it has substantially reduced spectral information. This is similar to what happens in cochlear implant signal processing. Time-domain signals from the experiments in this study for a particular clean vocoded speech utterance, a mixture of this clean vocoded speech and babble noise at −5 dB SNR, and the resulting vocoded speech signal using the estimated IBM have been illustrated in Figure 7. This was the same utterance as in Figure 6, but this time, the clean speech had been vocoded to mimic the behavior of a cochlear implant. This utterance was made up of eight words and the duration was just under three seconds. In the mixture, the clean speech was swamped by the babble noise and it would be extremely challenging for a hearing-impaired listener to discern what had been spoken. The estimated (enhanced) speech was not an exact replica of the clean speech but resembled it much more closely, and hence, there was a higher probability that the utterance could be understood.

Table 4 shows the intelligibility estimated using the NCM for the estimated masks for babble and SSN across a range of SNRs. It should be noted that all estimated masks performed well in terms of NCM improvement relative to the vocoded mixtures. The strongest performing mask for hearing-impaired speech intelligibility was the PSM+ while the least effective mask was the FFTM. When averaging over the two noise types and the three SNRs, the PSM+ showed a 27.8% improvement while the FFTM showed a 27.0% improvement. The PSM+ performed best for both babble noise and SSN and was closely followed by the QM.

### 5.5. Hearing-Impaired Speech Similarity

**Figure 8:**
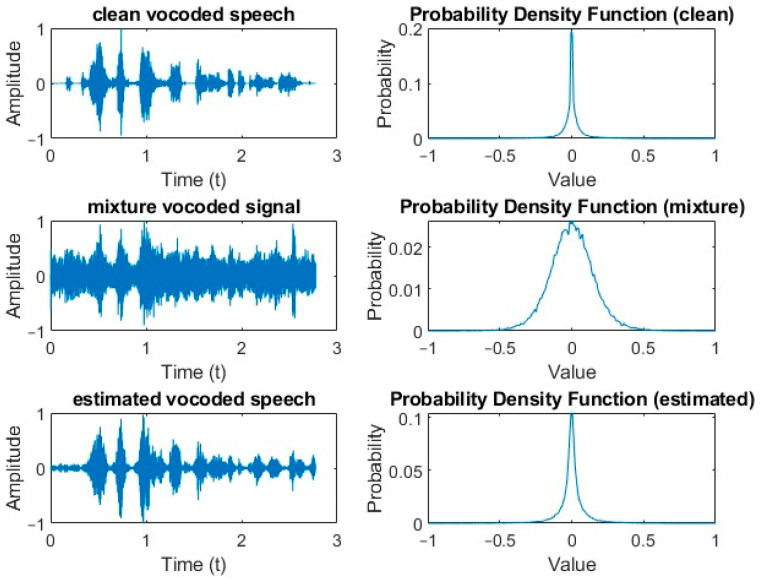
Time- domain representation and corresponding probability density functions of vocoded signals for clean speech, clean speech mixed with babble noise at −5 dB SNR, and IBM-estimated speech for the utterance “A plea for funds seems to come again”.

While it is not common practice to measure the quality of vocoded speech, it is useful to perform some comparison between clean vocoded speech, vocoded mixtures, and estimated vocoded speech. As discussed in Section 4.5, in this study, this was motivated by previous work on the use of “similarity” in speech and audio applications. The clean, mixture, and estimated vocoded signals were converted into histograms and then normalized so that they represented probability density functions, as shown in Figure 8. Similarity scores were calculated according to the methodology described in Section 4.5. The scores were separated according to the two noise types, i.e., babble and SSN. Each noise type was presented at three SNRs, as before. In general, the higher the similarity is, the better the score will be. The one exception to this is Shannon’s Entropy, where the smaller the Kullback–Leibler Divergence is, the greater the similarity will be. The results at the three different SNRs, for babble noise, are presented in Table 5, Table 6 and Table 7 while corresponding results for SSN are presented in Table 8, Table 9 and Table 10. 

#### 5.5.1. Babble Noise

Table 5 shows that the PSM+ and the QM had the highest similarity at −5 dB babble while the FFTM exhibited the lowest similarity. Table 6 shows that the QM had the best similarity at 0 dB babble while the FFTM exhibited the worst similarity. Table 7 shows that the IBM had the best similarity at 5 dB babble while the FFTM exhibited the worst similarity.

#### 5.5.2. Speech-Shaped Noise

Table 8 shows that the PSM+ and the QM had the best similarity at −5 dB SSN while the FFTM exhibited the worst similarity. Table 9 shows that the IBM had the best similarity at 0 dB SSN while the IRM exhibited the worst similarity. Table 10 shows that the PSM+ and the IBM had the best similarity at 5 dB SSN while the FFTM and the QM exhibited the worst similarity.

If the similarity/correlation scores are further averaged over both noise types and three SNRs, the best-performing mask is the QM with a score of 0.8508 and the least effective mask is the FFTM with a score of 0.8265. For reference, the corresponding score for the mixtures is 0.6421. If the KLD scores are averaged over both noise types and three SNRs, the best performing mask is the QM with a score of 0.0423 and the least effective mask is the FFTM with a score of 0.0648. For reference, the corresponding score for the mixtures is 0.4826.

## 6. Discussion

In order to set the results of this study in the context of the literature, the speech intelligibility results of Wang et al. [32] for normal speech are used as a basis for comparison. They used a similar neural network architecture to the one used in this paper. However, they used the TIMIT database for speech utterances, the NOISEX database for noise sources, a 64-channel Gammatone filterbank to generate the masks (as opposed to the STFT used here), and a set of complementary features. They also used the normalization and compression of unbounded mask values. However, [34] recommended that masks that are unbounded should not be compressed. Therefore, it was decided not to compress the masks in this study. 

Table 11 shows a comparison of the results from Wang et al. [32] for noisy speech and speech estimated using three masks (i.e., the masks common to both [32] and this study) with the results from this study for two noise types and three SNRs. The Pearson correlation between both sets of results was 0.992, which indicates a strong correlation. For this set of STOI scores, the mean over all mixtures, mask types, noise types, and SNRs for [32] was 0.754 versus 0.764 for this study. Correspondingly, the standard deviation for [32] was 0.098 versus 0.104 for this study. This would suggest that the short-time Fourier Transform (STFT) method, for generating the masks, results in marginally better STOI scores than using the Gammatone filter approach. 

In general, the PESQ scores of [32] were consistently higher than for this study but followed the same trend. When the speech utterances used in this study were resampled from 16 kHz (wideband) to 8 kHz (narrowband), the PESQ scores were closer to those of [32]. The objective measurement of speech quality is not considered as important as that of speech intelligibility for hearing-impaired listeners as it is more important to understand what is being said rather than how it is said [49]. Nonetheless, speech quality is still considered here as it is beneficial to be able to predict how comfortable it is for someone to listen to speech, especially in the presence of background noise. PESQ scores are used here to predict the quality of speech for normal speech only. The PSM+ is the best performing mask across both noise types over a range of SNRs for both STOI and PESQ.

When speech is noise vocoded to mimic the sound heard by the wearer of a cochlear implant, the masks newly proposed in this study perform best, namely the QM and the PSM+. When averaging over SNRs, PSM+ performs marginally better for both babble and SSN.

The Multilayer Perceptron architecture was used as the benchmark in this study. A different architecture might change the absolute performance but probably would not change the relative behavior of the different masks. To verify this, future work could be conducted using an alternative architecture such as the Deep Complex Convolution Recurrent Network (DCCRN) used in [65]. Employing such a complex network would allow for complex-valued spectrum modelling.

**Figure 9:**
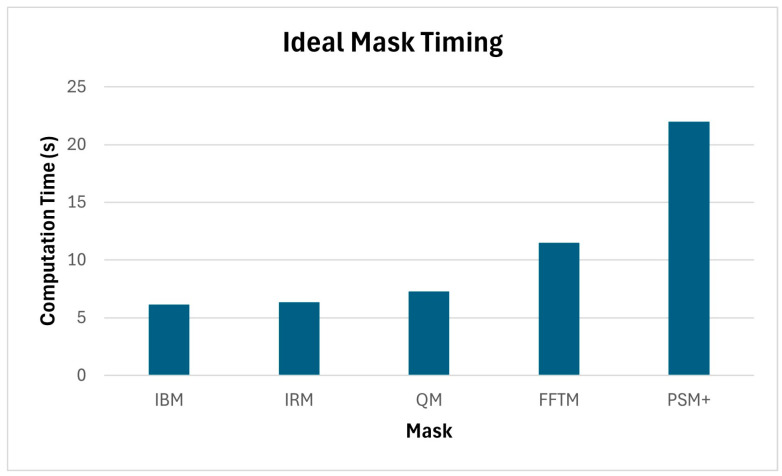
Time to generate training mixtures and compute ideal masks.

The masks were also compared in terms of their respective computational load based on a standard Windows laptop (Intel Core i7 processor) running MATLAB R2023b. The time was measured for how long it took to generate the 600 training mixtures and calculate the respective ideal masks. This was achieved using babble noise at −5 dB SNR. The results are illustrated in Figure 9. As expected, the IBM took the least amount of time (6.14 s) as it is the simplest mask. The IRM took slightly longer to calculate (6.33 s). The QM, which is one of the proposed new masks, took slightly longer at 7.27 s. This was followed by the FFTM at 11.49 s. The mask that took the longest time to calculate was the PSM+ at 22.00 s. The main reason for this was that in effect, two masks were being calculated, i.e., the PSM and the IRM. Also, the PSM involves the calculation of the cosine of a phase angle.

The similarity, correlation, and divergence values were measured for the vocoded mixtures and the vocoded clean speech utterances as a reference. Then, the same calculations were repeated for all five estimated masks, but this time between the estimated vocoded speech and the clean vocoded utterances. As expected, both similarity and correlation increased while divergence decreased for both noise types as the SNR was increased. The probability density functions (pdfs) are observed in Figure 8 for a particular clean vocoded utterance, the same vocoded utterance mixed with babble noise, and the IBM-vocoded estimate of speech. The mean values for the clean vocoded, vocoded mixture, and estimated vocoded signals were all approximately 0. However, their standard deviations were 0.0906, 0.1558 and 0.1028, respectively. This indicates that the standard deviations of the clean and estimated signals were fairly similar but significantly different from those of the vocoded mixture. When viewing the pdfs, the peaks of the clean vocoded, mixture vocoded, and estimated vocoded signals were 0.2, 0.025 and 0.1, respectively. The peaks of the clean and estimated vocoded were of the same order of magnitude but were significantly different from that of the vocoded mixture, resulting in the flatter nature of its pdf. Overall, the similarity results were consistent with other results regardless of which similarity family was used. 

All testing in this study used objective metrics to measure the performance of the masks for intelligibility and quality. Future research work might also be conducted to corroborate these results using subjective listening tests. For example, it would be of interest to compare the results of percent-correct word recognition scores and speech reception threshold to the NCM scores from Section 5.

## 7. Conclusions

The selection of an appropriate training target is important for both learning and generalization in speech separation [5]. This is particularly important when considering a speech enhancement system for cochlear implants. In this study, five different masks were compared using a feedforward deep neural network containing three hidden layers. Three of the masks had been well established in the literature and two were proposed in this work. All masks performed well for both speech quality and intelligibility for normal hearing. The proposed masks performed best in this regard. All masks performed well for intelligibility, similarity, and divergence for vocoded speech. The proposed masks also performed best in this case. Based on the literature and the results of this study, it is beneficial to combine both the magnitude and phase when designing a mask to be used as a training target in a noise reduction system for cochlear implants.

## Figures and Tables

**Table 1:** STOI scores for normal speech using oracle masks at −5 dB SNR for three noise types. ‘Mix’ indicates the unprocessed mixture (most and least effective scores are in bold and italics, respectively).

**Mask**	**Mix (babble)**	**IBM**	**IRM**	**FFTM**	**QM**	**PSM**
mean	0.5483	*0.8761*	0.9171	**0.9364**	0.8861	0.9343
median	0.5502	*0.8787*	0.9188	**0.9379**	0.8883	0.9356
standard deviation	0.055	*0.0213*	0.0156	0.0136	0.0199	**0.0134**
**Mask**	**Mix (SSN)**	**IBM**	**IRM**	**FFTM**	**QM**	**PSM**
mean	0.574	*0.872*	0.9114	**0.9321**	0.8825	0.9288
median	0.5789	*0.8746*	0.9128	**0.9338**	0.8846	0.9302
standard deviation	0.0524	*0.0211*	0.0157	**0.0143**	0.0196	**0.0143**
**Mask**	**Mix (factory)**	**IBM**	**IRM**	**FFTM**	**QM**	**PSM**
mean	0.5525	*0.8717*	0.9172	**0.937**	0.8821	0.935
median	0.5559	*0.8737*	0.9187	**0.9385**	0.8853	0.9367
standard deviation	0.0492	*0.023*	0.0149	0.013	0.0212	**0.0129**

**Table 2:** Mean STOI scores for normal speech using estimated masks at three SNRs for two noise types (most and least effective scores are shown in bold and italics, respectively).

Noise	SNR (dB)	Mask					
		Mix	IBM	IRM	FFTM	QM	PSM+
babble	−5	0.5599	0.6328	0.6333	**0.6396**	*0.6326*	0.6358
babble	0	0.6738	0.7717	*0.7659*	**0.775**	0.7693	0.7737
babble	5	0.7889	0.8684	*0.8639*	0.8677	0.8686	**0.8701**
SSN	−5	0.5849	*0.7231*	0.7256	0.7305	0.7231	**0.7307**
SSN	0	0.7047	0.8322	*0.8289*	0.8363	0.8328	**0.838**
SSN	5	0.8216	0.9043	*0.9017*	0.9072	0.9046	**0.9092**
MEAN		0.688967	0.78875	*0.78655*	0.792717	0.7885	**0.792917**

**Table 3:** Mean PESQ scores for normal speech using estimated masks at three SNRs for two noise types (most and least effective scores are shown in bold and italics, respectively).

Noise	SNR (dB)	Mask					
		Mix	IBM	IRM	FFTM	QM	PSM+
babble	−5	1.0692	*1.1004*	1.1195	1.1142	1.1038	**1.1348**
babble	0	1.0895	1.2511	1.2541	*1.2376*	1.2622	**1.2932**
babble	5	1.1978	1.6059	1.5391	*1.5191*	**1.6156**	1.5774
SSN	−5	1.0479	*1.1595*	1.1675	1.1723	1.1618	**1.1904**
SSN	0	1.0787	1.3317	*1.3161*	1.3365	1.3356	**1.3676**
SSN	5	1.1597	1.6618	*1.605*	1.6123	1.6704	**1.7079**
MEAN		1.107133	1.351733	1.33355	*1.332*	1.358233	**1.37855**

**Table 4:** NCM scores for vocoded speech using estimated masks at three SNRs for two noise types (most and least effective scores are shown in bold and italics, respectively).

Noise	SNR (dB)	Mask					
		Mix	IBM	IRM	FFTM	QM	PSM+
babble	−5	0.3651	*0.5385*	0.5492	0.5482	0.5448	**0.555**
babble	0	0.5626	0.7121	0.7137	*0.7109*	**0.7166**	0.7157
babble	5	0.7149	0.813	0.8091	*0.8063*	**0.8131**	0.8103
SSN	−5	0.4456	*0.701*	0.7101	0.7075	0.7036	**0.7126**
SSN	0	0.631	0.8018	0.7984	*0.7953*	**0.802**	0.8001
SSN	5	0.756	0.8469	0.8469	*0.8447*	**0.8469**	0.8468
MEAN		0.5792	0.73555	0.7379	*0.73548*	0.737833	**0.740083**

**Table 5:** Similarity scores for vocoded speech using estimated masks at −5 dB babble (most and least effective scores are shown in bold and italics, respectively).

Metric/Mask	Mix	IBM	IRM	FFTM	QM	PSM+
Lp	0.7765	0.8632	0.8673	*0.8585*	0.8714	**0.8716**
L1	0.6722	0.8401	0.8417	*0.8305*	**0.8485**	0.8412
Intersection	0.3339	0.5356	0.5389	*0.5243*	**0.5472**	0.5409
Inner Product	0.1999	0.4138	0.4228	*0.4082*	0.428	**0.431**
Fidelity	0.7441	0.9164	0.9189	*0.9115*	**0.9228**	0.9196
Squared L2	0.9376	0.9754	0.9763	*0.9735*	**0.9778**	0.9773
Shannon’s Entropy	0.5999	0.1164	0.1102	*0.1267*	**0.102**	0.1074
Combinations	0.6368	0.8544	0.8586	*0.8463*	**0.8649**	0.8612
Correlation	0.5724	0.8756	0.8754	*0.8572*	**0.8875**	0.8789

**Table 6:** Similarity scores for vocoded speech using estimated masks at 0 dB babble (most and least effective scores are shown in bold and italics, respectively).

Metric/Mask	Mix	IBM	IRM	FFTM	QM	PSM+
Lp	0.7891	0.9136	0.9023	*0.8889*	**0.9356**	0.9062
L1	0.717	0.8936	0.88	*0.8661*	**0.911**	0.8793
Intersection	0.3834	0.6097	0.5916	*0.5719*	**0.6364**	0.593
Inner Product	0.2452	0.4869	0.4744	*0.4563*	**0.5125**	0.4808
Fidelity	0.8017	0.9505	0.9431	*0.9353*	**0.9599**	0.9434
Squared L2	0.945	0.9889	0.986	*0.9827*	**0.9934**	0.9869
Shannon’s Entropy	0.4296	0.0479	0.0602	*0.0753*	**0.0322**	0.0591
Combinations	0.6991	0.9137	0.9008	*0.886*	**0.9332**	0.9026
Correlation	0.6418	0.9497	0.9317	*0.9134*	**0.972**	0.9353

**Table 7:** Similarity scores for vocoded speech using estimated masks at 5 dB babble (most and least effective scores are shown in bold and italics, respectively).

Metric/Mask	Mix	IBM	IRM	FFTM	QM	PSM+
Lp	0.8103	**0.9675**	0.929	*0.9281*	0.9672	0.9458
L1	0.7685	**0.9406**	0.9088	*0.9079*	0.9402	0.9208
Intersection	0.4434	**0.6793**	0.6311	*0.6297*	0.6788	0.6505
Inner Product	0.3084	**0.5416**	0.5045	*0.5035*	0.5414	0.5223
Fidelity	0.856	**0.9728**	0.9581	*0.958*	0.9726	0.9641
Squared L2	0.9555	**0.9982**	0.9921	*0.992*	0.9982	0.9951
Shannon’s Entropy	0.2717	**0.0152**	0.0354	*0.0357*	0.0154	0.0263
Combinations	0.7662	**0.9609**	0.9292	*0.9283*	0.9607	0.9427
Correlation	0.7275	**0.9954**	0.9643	*0.9642*	0.9953	0.9794

**Table 8:** Similarity scores for vocoded speech using estimated masks at −5 dB SSN (most and least effective scores are shown in bold and italics, respectively).

Metric/Mask	Mix	IBM	IRM	FFTM	QM	PSM+
Lp	0.767	0.8853	0.8814	*0.8796*	0.8945	**0.8949**
L1	0.6368	0.866	0.8564	*0.854*	**0.8744**	0.8579
Intersection	0.2954	0.5699	0.5592	*0.5562*	**0.5823**	0.5682
Inner Product	0.1651	0.4504	0.447	*0.4444*	0.4633	**0.4694**
Fidelity	0.6921	0.9339	0.9291	*0.9275*	**0.9391**	0.9321
Squared L2	0.9317	0.982	0.9804	*0.9798*	**0.9843**	0.9836
Shannon’s Entropy	0.757	0.0785	0.0866	*0.0897*	**0.068**	0.0779
Combinations	0.5831	0.8838	0.8766	*0.8742*	**0.8936**	0.8855
Correlation	0.5076	0.9117	0.8969	*0.8925*	**0.9238**	0.9134

**Table 9:** Similarity scores for vocoded speech using estimated masks at 0 dB SSN (most and least effective scores are shown in bold and italics, respectively).

Metric/Mask	Mix	IBM	IRM	FFTM	QM	PSM+
Lp	0.7813	**0.9645**	*0.9231*	0.9272	0.9641	0.9408
L1	0.6939	**0.9378**	*0.9022*	0.9063	0.9376	0.9101
Intersection	0.3573	**0.6755**	*0.622*	0.6278	0.6751	0.6386
Inner Product	0.22	**0.5379**	*0.4984*	0.5024	0.5372	0.52
Fidelity	0.7738	**0.9712**	*0.9545*	0.9567	0.971	0.9592
Squared L2	0.9404	**0.9978**	*0.9907*	0.9915	0.9977	0.994
Shannon’s Entropy	0.5127	**0.0169**	*0.0409*	0.0376	0.0171	0.033
Combinations	0.6674	**0.9583**	*0.9229*	0.9269	0.9581	0.9352
Correlation	0.5972	**0.9935**	*0.9572*	0.9615	0.9931	0.9733

**Table 10:** Similarity scores for vocoded speech using estimated masks at 5 dB SSN (most and least effective scores are shown in bold and italics, respectively).

Metric/Mask	Mix	IBM	IRM	FFTM	QM	PSM+
Lp	0.8012	0.9556	0.9512	*0.9473*	0.9518	**0.9637**
L1	0.7501	0.9312	0.9306	0.9279	*0.9276*	**0.9376**
Intersection	0.4216	0.6659	0.6621	*0.6578*	0.661	**0.6751**
Inner Product	0.2847	**0.5451**	0.5245	*0.5208*	0.5447	0.5369
Fidelity	0.8373	0.9704	0.9675	*0.9664*	0.9693	**0.9708**
Squared L2	0.951	0.997	0.9959	*0.9953*	0.9966	**0.9976**
Shannon’s Entropy	0.3248	**0.0179**	0.0221	*0.0236*	0.0191	**0.0179**
Combinations	0.7427	0.9523	0.9496	*0.9466*	0.9492	**0.958**
Correlation	0.6897	**0.9947**	0.9835	*0.981*	0.9941	0.9915

**Table 11:** STOI scores from Wang et al. [32] vs. this study.

**Mask**	**Noise**	**SNR**	**Wang et al.**	**This Study**
Mix	babble	−5	0.55	0.5599
IBM	babble	−5	0.63	0.6328
IRM	babble	−5	0.63	0.6333
FFTM	babble	−5	0.65	0.6396
Mix	SSN	−5	0.57	0.5849
IBM	SSN	−5	0.72	0.7231
IRM	SSN	−5	0.73	0.7256
FFTM	SSN	−5	0.74	0.7305
Mix	babble	0	0.66	0.6738
IBM	babble	0	0.76	0.7717
IRM	babble	0	0.76	0.7659
FFTM	babble	0	0.77	0.775
Mix	SSN	0	0.69	0.7047
IBM	SSN	0	0.82	0.8322
IRM	SSN	0	0.83	0.8289
FFTM	SSN	0	0.83	0.8363
Mix	babble	5	0.77	0.7889
IBM	babble	5	0.86	0.8684
IRM	babble	5	0.86	0.8639
FFTM	babble	5	0.85	0.8677
Mix	SSN	5	0.81	0.8216
IBM	SSN	5	0.87	0.9043
IRM	SSN	5	0.88	0.9017
FFTM	SSN	5	0.85	0.9072

## Data Availability

The dataset used in this study is publicly available and has been cited in Section 4.1 of this paper.
